# Significance of CT scan and color Doppler duplex ultrasound in the assessment of Abernethy malformation

**DOI:** 10.1186/s12880-015-0079-7

**Published:** 2015-09-18

**Authors:** Lucas Souto Nacif, Denise Cerqueira Paranaguá-Vezozzo, Flávio Henrique Ferreira Galvão, Manoel S. Rocha, Wellington Andraus, Flair Jose Carrilho, Luiz Carneiro D´Albuquerque

**Affiliations:** Liver and Gastrointestinal Transplant Division. Department of Gastroenterology, University of São Paulo School of Medicine, Rua Dr. Enéas de Carvalho Aguiar, 255 -9° andar -sala 9113/9114 CEP 05403-900, São Paulo, Brazil

**Keywords:** Abernethy malformation, Liver surgery, Liver transplantation, Abdominal imaging

## Abstract

**Background:**

Abernethy malformation is a rare congenital vascular abnormality in which the portal vein bypasses the liver and drains directly into the inferior vena cava. Diagnosis is complex and requires good quality imaging methods to identify details in systemic and portal circulation in order to establish diagnostic confirmation and treatment strategy. In this study we highlight the significance of the use of CT scans and Color Doppler Duplex Ultrasound for the diagnosis, treatment and evolution assessment in two adults with Abernethy malformation.

**Case presentation:**

The diagnosis and the treatment of two patients with Abernethy malformation by CT scan and Color Doppler Duplex Ultrasound is described. One patient was submitted to liver transplantation due to chronic liver disease and multiple nodules diagnosed as adenoma. The other patient had normal liver function and a mild neurological and psychomotor dysfunction, therefore we adopted clinical treatment and close liver parenchyma evaluation and nodule surveillance, using an imaging approach involving intercalating CT scan and Color Doppler Duplex Ultrasound every 6 months. We highlight some important direct and indirect findings of non-invasive imaging methods.

**Conclusion:**

Abernethy malformation requires meticulous image diagnosis to improve treatment and avoid iatrogenic procedures. CT scans and Color Doppler Duplex Ultrasound are both efficient methods for diagnosis, treatment planning and evolution assessment of patients with Abernethy malformation.

## Background

Abernethy malformation (AM) is a rare congenital abnormality described by John Abernethy in 1793. It is characterized by a shunt between the portal vein and systemic circulation [1, 2]. It is frequently associated with other congenital abnormalities, including the absence of a portal vein and/or congenital mesenterico-caval shunt, cardiac and/or pulmonary malformation and liver nodules [3, 4]. Complications of Abernethy malformation include hepatic encephalopathy, hepatopulmonary syndrome, portopulmonary syndrome and hepatic tumors [2].

**Fig. 1:**
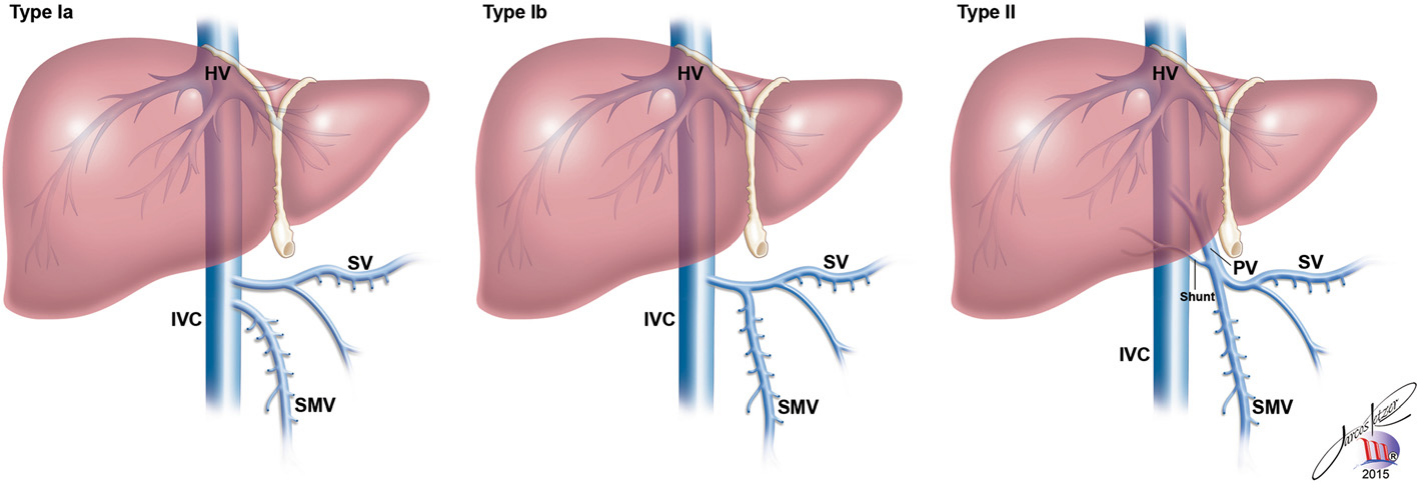
Classification of portosystemic anomalies (Abernethy malformation): Type I: Liver not perfused with portal blood - complete end-to-side shunt. Ia: Superior mesenteric vein and splenic vein do not join to form a confluence congenital absence of the portal vein. Ib: Superior mesenteric vein and splenic vein join to form a confluence portal vein. Type II: Liver perfused with portal blood - partial side-to-side shunt

There are two types of AM demonstrated on Fig. 1. Type I is defined as a complete porto systemic shunt, in which the portal vein merges with the inferior vena cava (IVC) in an end-to-end fashion, resulting in a complete absence of extra hepatic portal vein flow to the liver. Type I is further sub classified into types Ia and Ib according to the course of the splenic and mesenteric veins. AM type II refers to a partial shunt, consisting of a side-to-side connection between the portal vein and the systemic venous circulation, with a small degree of portal flow to the liver [1–4].

A meticulous assessment of the liver and vascular anatomy by non-interventional abdominal exams in Abernethy malformation aids diagnosis and planning of appropriate treatment. In this study, we detailed some important direct and indirect findings of non-invasive imaging methods and highlight the value of CT scans and Color Doppler Duplex Ultrasound for the diagnosis, treatment and evolution assessment of two adult patients with Abernethy malformation.

## Case presentation

**Fig. 2:**
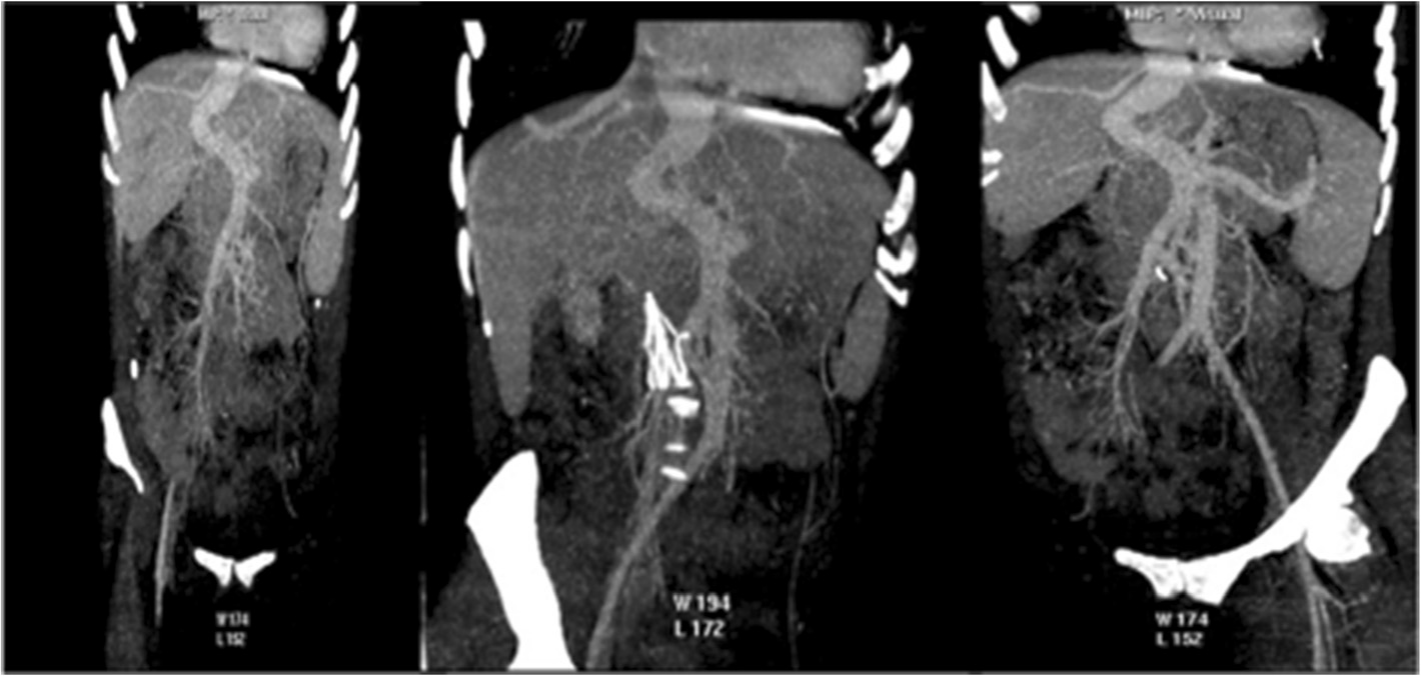
CT coronal planes in arterial and portal phases on case 1 (Type 1 – end-to-end). Note: Direct portal systemic shunt with evidence of the longitudinal path along the upper mesenteric vein, portal vein axis with the deviation and a kinking route could be observed. Without any hepatic vein intra and finishing in inferior cava vein near the right atrium

**Fig. 3:**
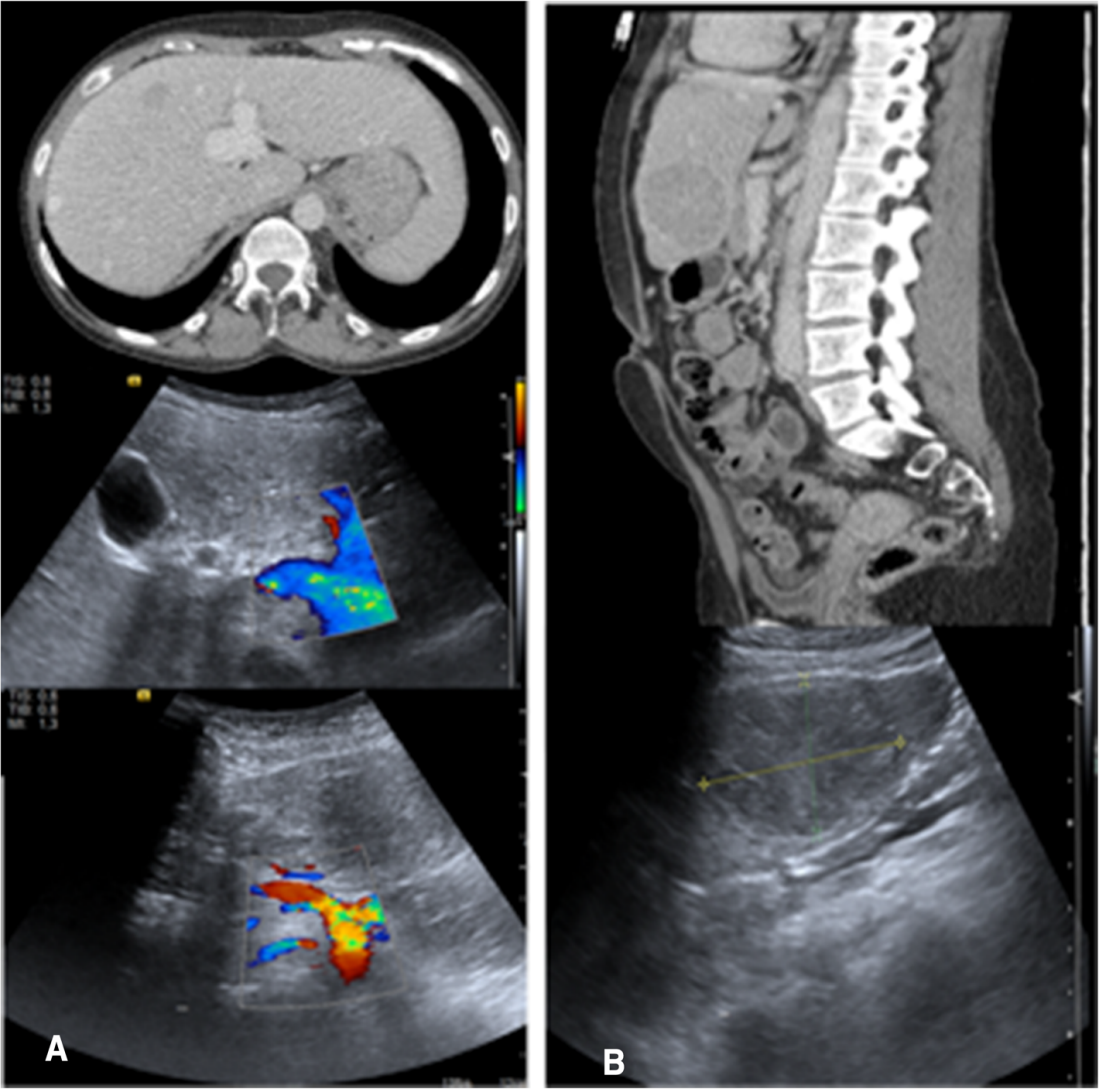
Correlation between CT and Color Doppler Duplex Ultrasound in axial and sagittal planes. Note: Case 1 (Type 1) **a**: Axial CT plane in the venous ligament level with left branch of portal vein extra hepatic dilated. Color Doppler Duplex US below shows us an extra hepatic portal route and a dilated hepatic artery emerging from the celiac trunk. **b**: Sagittal planes show a hypodense lesion in segment III (50 X40 mm) and predominantly hypoechoic, respectively at TC and the US

The first patient was a 20-years-old female with chronic renal failure and systemic arterial hypertension, who had been on dialysis since February 2012 and on the waiting list for kidney transplantation. She had heart surgery at the age of 8 to treat a congenital heart disease (CIA type ostium secundum) and a disabled factor XII of 20 % (thrombotic factors predisposes). She also presented delayed psychomotor and learning development. Clinical examination showed no external phenotypic malformation evidence. In genetic assessment, we found a heterozygous A1298C MTHFR gene mutation. During abdominal investigation for renal transplantation, an ultrasonography and CT scan detected chronic liver disease, multiple nodules and the portal vein with a regular splenomesenteric junction path, but draining directly into the inferior vena cava, close to the right atrium as described in the pathology of Type 1 Abernethy malformation, demonstrated in Fig. 2. The main portal trunk and a large hepatic artery at the liver’s hilum were absent. A liver biopsy was performed on a large hypoechoic lesion in segment III (50 X40 mm), which was diagnosed as adenoma without malignant cells, shown in Fig. 3. The liver enzymes were close to the normal values in assessment, but the echocardiography showed a hypertensive cardiomyopathy and mild aortic mitral regurgitation. This patient underwent combined liver and kidney transplantation in July 2014, and is currently presenting favorable clinical conditions and normal laboratorial analysis.

**Fig. 4:**
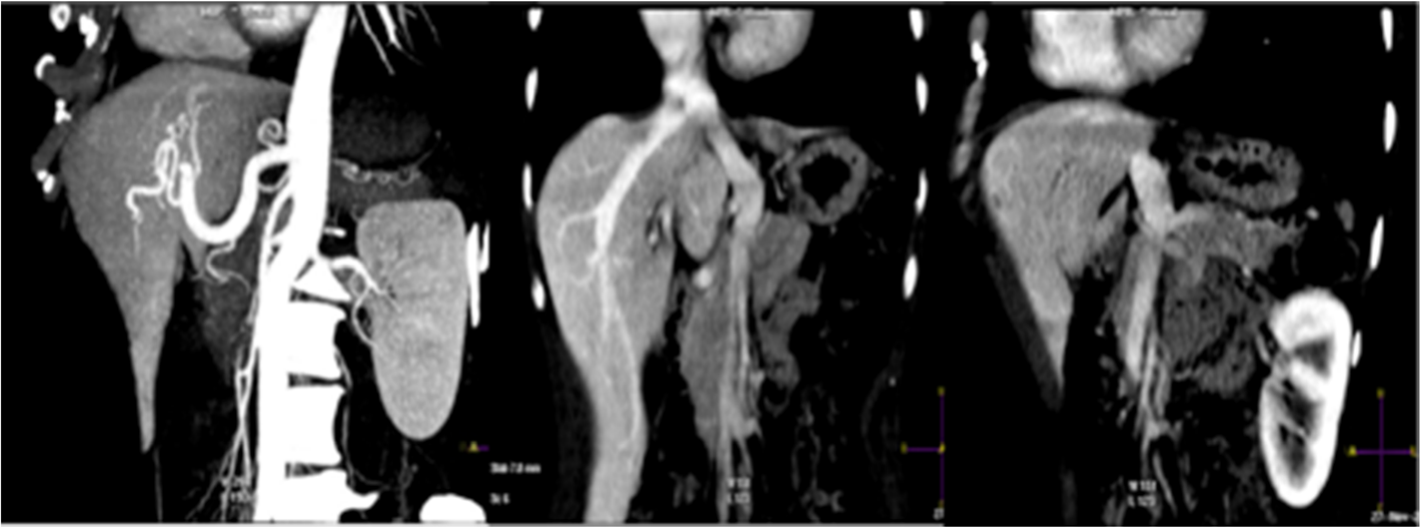
CT coronal planes in arterial and portal phases on case 2 (Type 2-side-to side). Note: The abdominal aorta with an increased caliber of the hepatic artery and its intrahepatic branches hypertrophied. Confluence of the middle hepatic vein and the extra hepatic portal vein to the inferior vena cava

**Fig. 5:**
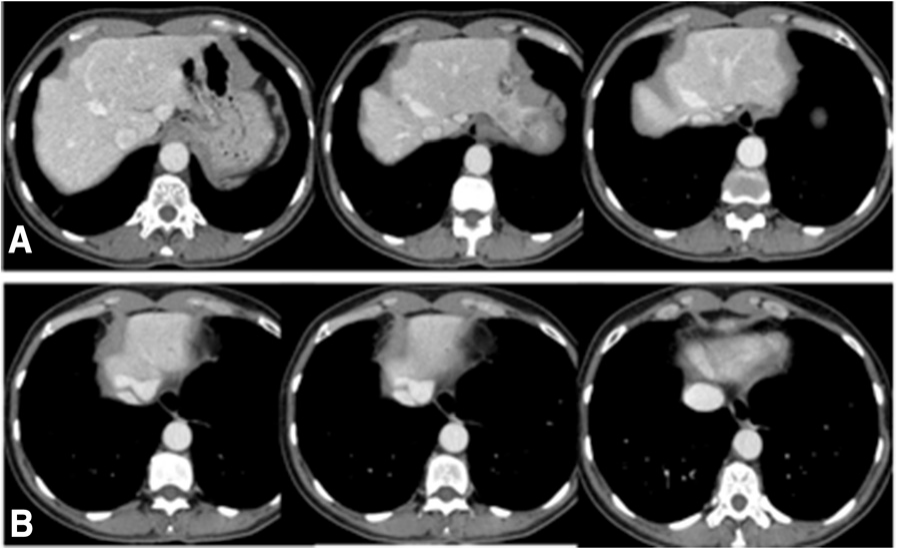
Abdominal CT with dynamic study in the portal phase in axial plane on case 2 (Type 2). Note: **a**: axial plane on the topography of venous ligament, highlighting the vascular route along the caudate segment and converging path of hepatic veins to the inferior vena cava. Featured of the extrahepatic portal vein hinting at the venous ligament. **b**: In the most cranial axial liver, showing the junction of the extrahepatic portal vein with the medial aspect of the inferior vena cava

**Fig. 6:**
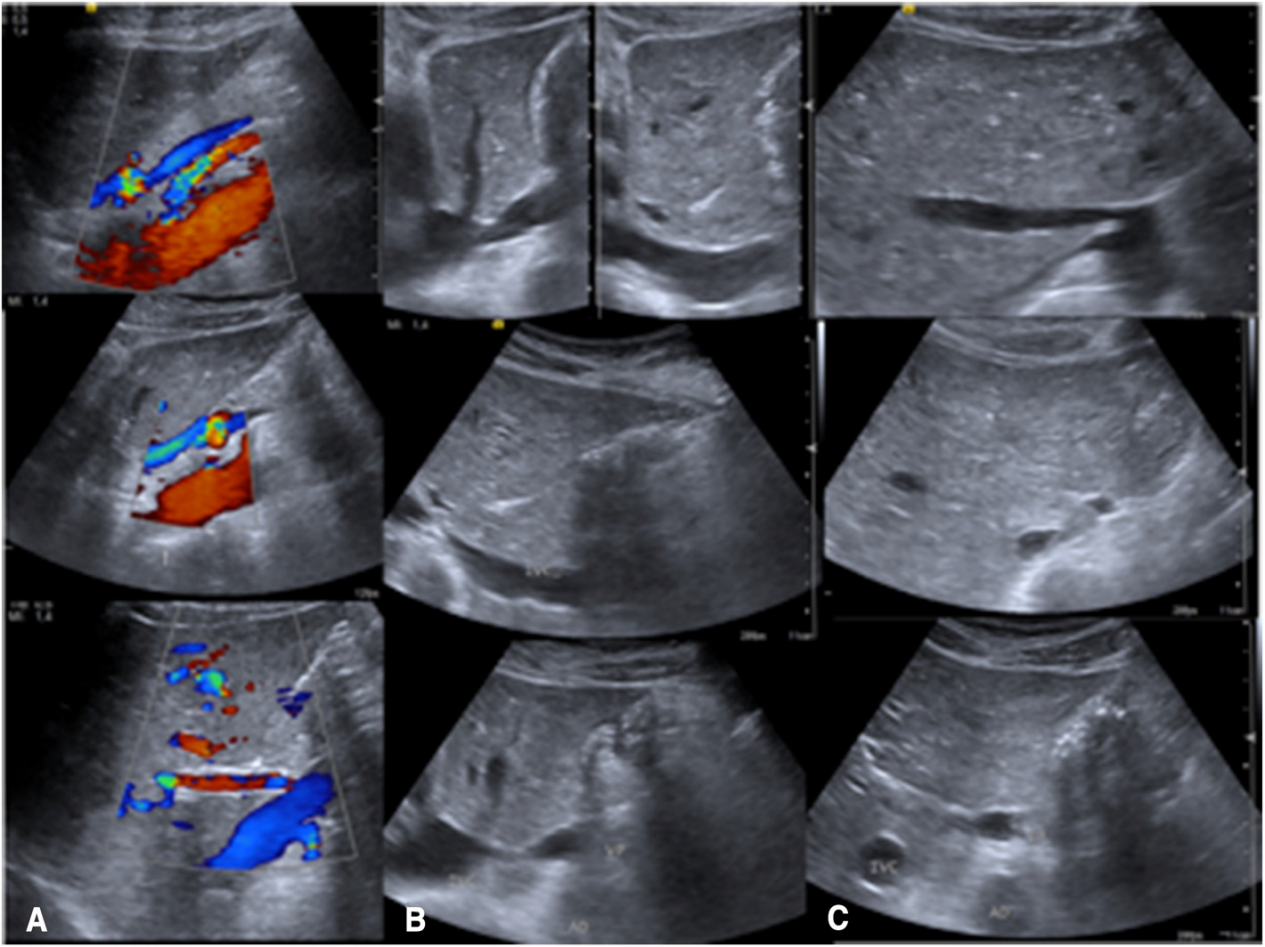
Color Doppler Ultrasound with sagittal and axial planes on case 2 (Type 2). Note: **a**: The abdominal aorta, mesenteric artery, extension of mesenteric vein and also the extrahepatic portal vein that directs to the head and posterior region of the lateral segment. At hepatic hilum level there is an evidence of the inferior vena cava and an increased caliber of hepatic artery. **b**: The sagittal and paramedian planes show two retro hepatics paths of the portal extrahepatic vein (along the venous ligament). **c**: Ultrasound in axial plane, in the level of the confluence of the hepatic veins to IVC and cross-section of the inferior vena cava, the extrahepatic portal vein door and the abdominal aorta

In the second case, the patient was a 52-year-old female with celiac disease and hypothyroidism, diagnosed 10 years ago. Initially asymptomatic, AM was first suspected after a routine ultrasound exam six years ago for abdominal pain, at the time diagnosed as gastritis. The angio CT (Fig. 4) clearly showed extra portal vein drainage into the inferior vena cava, above the liver, characteristic of Type 2 (side-to-side) Abernethy malformation, and intra-liver arteriovenous shunts (Fig. 5). In the Color Doppler Ultrasound (Fig. 6) exam, we found higher intrahepatic arterialization and portal vein malformation because of the fusion of the mesenteric vein with the portal vein, above the hilum of the liver entrance, where the path follows the venous ligament. She is stable, under clinical surveillance since 2009, however in a recent assessment, the patient showed impaired frame memory and attention span. In neurological and psychomotor evaluation she presented averbal episodic memory deficit with a delayed recall. The functioning and coordination of fine motor and the processing speed that she presented could be related to metabolic disorders and hearing impairment. Because of these findings, she was referred for speech therapy and neuropsychological rehabilitation, with promising improvements. All clinical evaluation, including gastrointestinal tract analysis and tumor markers were normal. The patient has refused liver transplantation, therefore clinical treatment and a close evaluation of the liver has been adopted, using an imaging approach intercalating CT scan and Color Doppler Duplex Ultrasound, mainly for liver nodule surveillance at six monthly intervals.

### Ethical aspects

This study was approved by the Institutional Review Board fulfilling all requirements for studies in humans, following the guidelines of the 1975 Declaration of Helsinki.

## Conclusions

AM is an extremely rare syndrome, with less than 20 cases reported in English language medical literature. Most patients with AM suffer premature mortality as a result of the serious conditions related to this syndrome. In those patients with mild conditions, the presence of a portocaval systemic shunt is often not recognized during childhood [5]. This diagnosis was also not recognized in infancy for these cases, but with appropriate imaging exams, accurate diagnosis was made in adulthood. The majority of affected patients already described were below the age of 18 and female [4]. Almost all reported cases were admitted to hospital with a variety of symptoms, including nausea, vomiting, fatigue, epigastric pain, asthenia, anorexia, jaundice and dyspnea [4].

The second case was initially presumed to be agenesis of the portal vein with decompensated hepatic dysfunction. However, after sagittal and coronal scans beyond the axial planes of the abdominal multidetector tomography, and detailed Doppler Duplex liver ultrasonography exams, the abnormal liver vascular anatomy was identified as AM. Occasionally, the image diagnosis is easy to make [6], but in this case, meticulous evaluation of the liver and vascular anatomy by non-interventional abdominal exams for Abernethy malformation with many plane sections improved the diagnosis.

Portal vein thrombosis exclusion is also a differential diagnosis of AM. Currently, cavernous transformation of chronic portal thrombosis can be identified with high accuracy using Doppler Duplex Ultrasonography exams and CT imaging [5]. The diagnosis of Abernethy malformation from acquired causes of absent portal veins depends on delineating of the extrahepatic portal vein anatomy by radiological exams, intraoperatively, or at autopsy. In this study, accurate diagnosis was made using non-invasive imaging methods without radiation, when the Doppler Duplex could indicate the portocaval shunt abnormality, or by angio-TC that could clarify the vascular anatomy instead of more invasive angiography exams [7]. In the first case the confirmation for diagnosis was achieved by the explant following liver transplantation.

The combination of both imaging methods, first US and than CT gives a better chance of detecting rare liver disease, considering also, a recently revision that suggests the US as the first line option to assess liver disease [8]. We showed (Figs. 2, 3 and 4) images changes along many slices and multiples scan where we could suggest the presence of vascular anomalous path through the venous ligament as an important direct signal effect. On the other hand, the indirect effects can be related to absence of a trunk portal vein through the hepatic hilum and along the right and left portal branch way (2^nd^, 3^rd^ and 4^th^ branch portal ramification, usually visualized in the conventional imaging methods), as the enlarged and hypertrophied hepatic artery in the hepatic hilum.

The main advantages of US are that it is non-invasive, available in most health centers, and is not expensive [8]. However, it is somewhat limited in its diagnostic accuracy by the fact that it depends heavily upon the opinion of the operator, and provides only a partial view of the anatomy in each scan. This makes it well suited to early identification or front line use, but diagnosis should be clarified with CT. The advantages of CT are that it is less subjective and provides a better panoramic view of the anatomy. However it is much more expensive and less commonly found in health centers, meaning that we recommend it as a reinforcement of the initial impression given by US.

The association of portal vein anomaly and nodular liver lesions was observed in almost half of the reported cases, and the most frequent lesion is the focal nodular hyperplasia [4]. The treatment options for Abernethy malformation include surgical correction of shunts, liver nodule resection and liver transplantation [4]. In these cases, the first one underwent liver transplantation and the second, was monitored by close clinical and imaging follow up.

In summary, we reported and detailed some important direct and indirect findings of non-invasive imaging methods. On two cases of AM in an attempt to evaluate the significance of CT angiography and Duplex Doppler liver plane anatomy to identify variations of liver anatomy and vascular malformations. These imaging methods are crucial to improve the clinical diagnosis of AM and to avoid iatrogenic procedures when planning liver surgery strategy.

## Consent

Written informed consent was obtained from both patients for publication of this Case report and any accompanying images. A copy of the written consent is available for review by the Editor of this journal.

## Abbreviation

CT: Computed tomography
AM: Abernethy malformation
IVC: Inferior vena cava
PV: Portal vein
SV: Splenic vein
SMV: Superior mesenteric vein
